# Enzymatic Biosynthesis of Simple Phenolic Glycosides as Potential Anti-Melanogenic Antioxidants

**DOI:** 10.3390/antiox11071396

**Published:** 2022-07-19

**Authors:** Hogwuan Jung, JaeWook Oh, Younghae Kwon, Woongshin Kang, Minsuk Seo, Yurin Seol, Je Won Park

**Affiliations:** 1 Transdisciplinary Major in Learning Health Systems, Department of Integrated Biomedical and Life Sciences, Korea University, Seoul 02841, Korea; dkslejtmzm@korea.ac.kr (H.J.); wook369@korea.ac.kr (J.O.); rnjsdudgo21@korea.ac.kr (Y.K.); dnd4730@korea.ac.kr (W.K.); panda7894@korea.ac.kr (M.S.); dlscjsdbfls@korea.ac.kr (Y.S.); 2 School of Biosystems and Biomedical Sciences, Korea University, Seoul 02841, Korea; 3 Department of Integrated Biomedical and Life Sciences, Korea University, Seoul 02841, Korea

**Keywords:** simple phenolic glycosides, glycosyltransferase, StSPGT, 4-hydroxyphenyl-2-propanoyl-*O*-α-glucoside, anti-melanogenic antioxidants, elastase inhibitor

## Abstract

Simple phenolics (SPs) and their glycosides have recently gained much attention as functional skin-care resources for their anti-melanogenic and antioxidant activities. Enzymatic glycosylation of SP aglycone make it feasible to create SP glycosides with updated bioactive potentials. Herein, a glycosyltransferase (GT)-encoding gene was cloned from the fosmid libraries of *Streptomyces tenjimariensis* ATCC 31603 using GT-specific degenerate PCR followed by in silico analyses. The recombinant StSPGT was able to flexibly catalyze the transfer of two glycosyl moieties towards two SP acceptors, (hydroxyphenyl-2-propanol [HPP2] and hydroxyphenyl-3-propanol [HPP3]), generating stereospecific α-anomeric glycosides as follows: HPP2-*O*-α-glucoside, HPP2-*O*-α-2″-deoxyglucoside, HPP3-*O*-α-glucoside and HPP3-*O*-α-2″-deoxyglucoside. This enzyme seems not only to prefer UDP-glucose and HPP2 as a favorable glycosyl donor and acceptor, respectively but also differentiates the positional difference of the hydroxyl function as acceptor catalytic sites. Paired in vitro and in vivo antioxidant assays represented SPs and their corresponding glycosides as convincing antioxidants in a time- and concentration-dependent manner by scavenging DPPH radicals and intracellular ROS. Even compared to the conventional agents, HPP2 and glycoside analogs displayed improved tyrosinase inhibitory activity in vitro and still suppressed in vivo melanogenesis. Both HPP2 glycosides are further likely to exert the best inhibitory activity against elastase, eventually highlighting these glycosides with enhanced anti-melanogenic and antioxidant activities as promising anti-wrinkle hits.

## 1. Introduction

**Figure 1:**
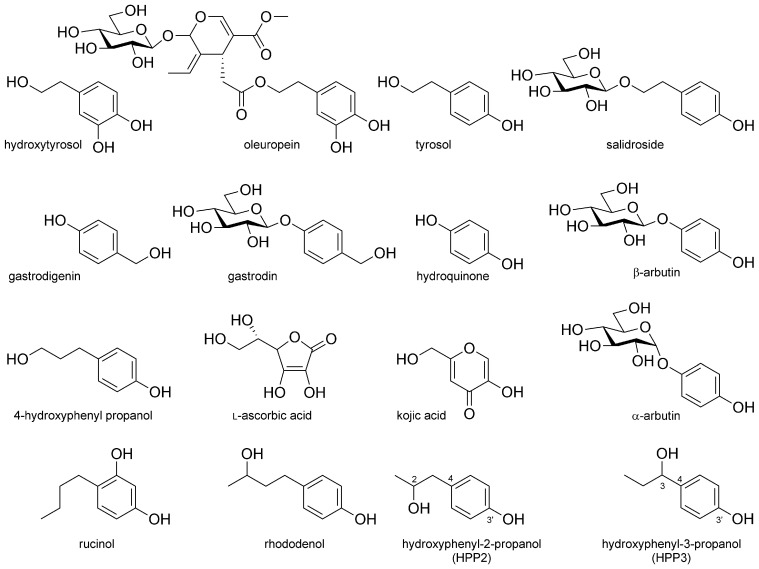
Chemical structures of simple phenolics, their glycosides and anti-melanogenic chemicals described in this study.

Simple phenolics (SPs) belong to phytochemicals that exist in a variety of forms in the kingdom of Plantae and are low-molecular-weight biomaterials that possess a wide range of biologically beneficial properties [1]. For instance, hydroxytyrosol (or 3,4-dihydroxyphenyl ethanol; depicted in Figure 1), which exists as a main component in olive leaves or processed olive oil, and its glycoside oleuropein (Figure 1) are reported to show, at the in vitro level, antioxidant activities that prevent oxidation of fats and nucleic acids, antimicrobial activities against some microorganisms, platelet aggregation inhibitory activities and endothelial dysfunction preventative activities [2,3,4,5]. Moreover, tyrosol (or 4-hydroxyphenyl ethanol; depicted in Figure 1), which is a sort of SP that has long been used as an herbal medicine in China, and *Rhodiola rosea* L. or Tibetan ginseng, which contains salidroside (or tyrosol glucoside; depicted in Figure 1), provide diverse physiological activities, such as antioxidant activities, cardiovascular disease preventative activities, antitumor and anti-fatigue activities, anoxia inhibitory activities and adaptogenic effects such as anti-aging activities [6,7]. In addition, gastrodigenin (or 4-hydroxybenzyl alcohol; depicted in Figure 1), as a major SP component inside *Gastrodia elata* Blume, is used as an herbal medicine, and gastrodin (as its glucoside; depicted in Figure 1) also present anti-convulsive improvements and neuroprotective activities as well as antioxidant abilities [8,9]. Accordingly, gastrodin is now used in clinical settings in China as a supporting drug to treat neurasthenia, headache and epilepsy [10]. On the other hand, hydroquinone (or 4-hydroxyphenol; depicted in Figure 1) and its glucoside β-arbutin (Figure 1), which are mainly extracted from bearberry, as well as 4-hydroxyphenyl propanol (Figure 1), exist as extracts from the leaves of *Pinus sylvestris* or Scotch pine and have been known for their antioxidant, anti-inflammatory, antitumor and skin-whitening activities [11,12,13]. Hence, the above-described SPs and their glycosides have recently garnered much attention as functional cosmetic bioresources for their anti-melanogenic and antioxidant activities.

Human skin color is mainly determined by the amount and distribution of melanin that exists in the epidermis. While melanin exists in the skin as a type of polymer combined with protein (it absorbs a wide range of lights, including ultraviolet rays, and therefore plays an important role in protecting the physical body from ultraviolet radiation), it could cause skin lesions such as vitiligo if its biosynthesis is impaired abnormally. Conversely, if melanin is overproduced, not only does it form freckles, melasma and skin hyperpigmentation but it is also known to exacerbate skin-aging and be closely related to skin cancer. Melanin is biosynthesized through a series of oxidation–reduction reactions of various enzymes including tyrosinase. Tyrosinase, which uses L-tyrosine as a substrate to convert it into L-DOPA (L-3,4-dihydroxyphenylalanine) and subsequently into L-DOPAquinone, is very important as the initial reaction to determine the pace of melanin biosynthesis [14,15]. In order to treat or mitigate abnormal skin pigmentation symptoms and the deposition of excessive melanin pigments caused by exposure to ultraviolet rays, a number of organic chemicals showing various tyrosinase inhibitory activities have been combined within cosmetic and medicinal formulations since the 1990s. L-Ascorbic acid (Figure 1) and its derivatives, such as kojic acids (Figure 1), are combined with cosmetics for their antioxidant activities against tyrosinase and for their chelating properties to copper atoms in metalloenzyme tyrosinase. In addition, α-arbutin (manufactured by Shiseido; depicted in Figure 1) and rucinol (4-butyl-benzol-1,3-diol manufactured by POLA; depicted in Figure 1) have been included in functional cosmetics as anti-melanogenic ingredients for their competitive inhibition activities of tyrosinase [16,17]. On the contrary, rhododenol (4-hydroxyphenyl-2-butanol; depicted in Figure 1) was manufactured by Kanebo but is currently banned for causing vitiligo [18]. The SP biochemicals structurally resemble the abovementioned cosmetic representatives with anti-melanogenic activities. According to recent publications, *Rhodiola rosea* L extracts, which contain the natural SP chemical tyrosol and its glucoside salidroside, are reported to have skin-whitening activities [19,20]. In addition, there have been reports about the inhibitory abilities on melanin biosynthesis of hydroxytyrosol derived from olive oil [21]. When it comes to natural products, however, difficulties harvesting them due to their unique climate characteristics, indiscriminate plant collecting and logging and difficulties in terms of low content and purification could be considered as obstacles to their industrial commercialization.

In the meantime, the SP aglycones tend to display advances in their in vivo absorption and metabolism rates, and when they are combined as formulations, their chemical stability is known to decline. However, when it comes to their corresponding glycosides, although their bio-absorption rate is not as high, their solubility and bioavailability can be enhanced more than aglycones [1,3,16,18]. In this regard, several studies [22,23,24,25,26,27,28] in which various organic or enzymatic synthesis methods were employed have consistently reported the manufacture of a series of SP glycosides. Enhanced bioactivities through structural modifications to the parental molecules are recognized as a feasible tool for the reformulation of the dosage forms of cosmetics and medicines; the enzymatic biosynthesis of glycosides using glycosyltransferase (GT) is capable of transferring the sugar moiety from the nucleotide activated glycosyl donors onto the hydroxyl function within the target aglycones (http://www.cazy.org; accessed on 1 July 2022). As the glycosylation of SP aglycones using the regio- and stereo-specific GT allows the biosynthesis of particular SP glycosides with enhanced solubility and stability together with extended bioavailability, when being administered in vivo (more so than the parental aglycones), these bioactivity advances facilitate the development of prodrugs and/or incrementally modified drugs. Therefore, we reasoned that it would be possible to discover new bacteria-origin GT specific to the glycosylation of SPs, which would thereby provide a clue for the biosynthesis of previously unprecedented (or unnatural) SP glycosides with enhanced bioactivities. This concept started with the screening of fosmid-based libraries for mining the GT-encoding gene candidates.

Herein, we aimed to isolate an SP GT-encoding gene from the formerly established fosmid libraries of soil actinomycetes *Streptomyces tenjimariensis* ATCC 31603 by employing GT-specific degenerate PCR primers. Along with in silico analyses of the expected protein followed by in vitro kinetic characterization, this glycosyltransferase StSPGT was expressed in *E. coli* BL21(DE3) as a hexa-histidine (His_6_)-tagged recombinant protein. In addition, the biosynthetic tailoring of the new SP glycosides, the chemical structures of which have not been previously observed, were performed through the enzymatic reaction of the recombinant StSPGT with substrates (including SP acceptors and nucleotide-activated glycosyl donors), and then their biological activities (including cytotoxicity, antioxidant, anti-melanogenic and elastase inhibitory properties) were comparatively evaluated.

## 2. Materials and Methods

### 2.1. Chemicals and Reagents

Hydroxyphenyl-2-propanol (HPP2) and hydroxyphenyl-3-propanol (HPP3) (as equimolar racemic mixtures; Figure 1) were purchased from Aurora Fine Chemicals (San Diego, CA, USA). Details regarding others are described in Supplementary Materials.

### 2.2. Isolation and Heterologous Expression of Recombinant StSPGT in E. coli

Bacterial strains, culture conditions, expression and purification of protein are described in Supplementary Materials.

### 2.3. In Vitro Enzymatic GT Reactions and Its Kinetic Studies

Details regarding StSPGT’s enzymatic reactions and its Michaelis–Menten kinetic analyses are described in Supplementary Materials.

### 2.4. Instrumental Analyses

Details regarding the instrumental analyses together with the chromatographic isolation and chemical characterization of biocatalytic products obtained are described in Supplementary Materials.

### 2.5. Biological Assays

The cytotoxic effects of SP aglycones and their glycosides on B16F10 cells was determined using a conventional MTT assay. B16F10 murine melanoma cells were routinely maintained in Dulbecco’s modified Eagle’s medium (DMEM) (Gibco, New York, NY, USA) supplemented with 10% heat-inactivated fetal bovine serum (Gibco), 100 units/mL penicillin and 100 μg/mL streptomycin (Gibco) at 37 °C under humidified 5% CO_2_ atmosphere. For the assay, these B16F10 cells were seeded in advance in 24-well plastic tissue culture plates at a density of 2 × 10^3^ cells/well. After 24 h cultivation, the cells within individual wells were treated with the indicated concentrations of SPs and their glycosides for 48 h. The cells were incubated with MTT solution for additional 4 h in the dark, and the reduced formazan crystals were then dissolved in DMSO; the DMSO percentage in the media was kept below 0.5%. The resulting mixture was transferred to a 96-well microplate, and then the absorbance was measured at 570 nm using a SpectraMax microplate reader (Molecular Devices, Sunnyvale, CA, USA).

DPPH (1,1-diphenyl-2-picryl-hydrazyl-hydrate) free radical scavenging assay was employed for determining the in vitro antioxidant activity of SP aglycones and their generated glycosides; L-ascorbic acid was utilized as a positive control whereas DMSO only was used as negative control. The antioxidant activities were expressed as IC_50_ value (as the concentration of an antioxidant at which 50% of inhibition of free radicals). In brief, 1.9 mL of the 100 μM DPPH reagent (in MeOH) was added into 0.1 mL of each sample diluted within 0.1 to 2 mM range (0.1, 0.2, 0.5, 1.0 and 2.0 mM dissolved in DMSO). After being mixed well by shaking for 5 s, the mixtures were then incubated at 30 °C for 15 min, followed by measuring absorbance with a microplate reader at 515 nm.

The intracellular reactive oxygen species (ROS) level was quantitatively determined using the dichlorofluorescin diacetate (DCFDA) cellular ROS detection assay kit (ab113851, Abcam, Cambridge, UK), according to the manufacturer’s instructions. Briefly, B16F10 cells were seeded in a 24-well plate at a density of 2 × 10^3^ cells/well. The cells were washed three times using PBS and subjected to staining with 20 μM DCFDA in PBS for 20 min at 37 °C under dark conditions. After staining, the cells were washed with PBS again and incubated with 50 μM TBHP solution (Sigma-Aldrich, St. Louis, MO, USA). Cells were treated time-dependently (10, 30 min and 1 h) with the indicated concentrations of SPs and their glycosides in 0.1% bovine serum albumin containing culture media after DCFDA incubation. The ROS level was analyzed by a fluorescence microplate reader (SpectraMax Gemini XPS/EM, Molecular Devices, Sunnyvale, CA, USA) set at an excitation wavelength of 485 nm and an emission wavelength of 545 nm. Relative fluorescence intensity of controls was equated to 100%, with treatment conditions estimated proportionally.

The in vitro inhibitory effects of SPs and their glycosides towards tyrosinase (Sigma-Aldrich; mushroom origin; 2000 Unit/mL) activities were determined using L-tyrosine as a substrate. Briefly, the reaction mixtures, comprising 100 μL phosphate buffer (pH 6.8, 100 mM), 20 μL of tyrosinase (250 U/mL) and 20 μL of varying concentrations of SPs and their glycosides (in DMSO), were preincubated at 30 °C for 10 min. After pre-incubation, 20 μL of 1.5 mM L-tyrosine was added into each well in a 96-well microplate and further incubated for 10 min. The absorbance of each resulting reactant was measured with a microplate reader at 475 nm, and tyrosinase inhibitory activities were expressed as IC_50_ values (as a concentration of an enzyme inhibitor that caused 50% tyrosinase activity inhibition) by interpolation of the dose–response curves; kojic acid (Sigma-Aldrich) and hydroquinone (Sigma-Aldrich), being comparable, were used as positive controls, whereas DMSO only was used as negative control.

The inhibitory effects of SPs and their glycosides towards cellular melanin biosynthesis were examined using the above-described B16F10 murine melanoma cells. After 24 h seed cultivation in a 24-well plate, these cells were treated with the indicated concentrations of SPs and their glycosides for a further 48 h in the presence of 2 μM α-melanocyte stimulating hormone (α-MSH, Sigma-Aldrich). After washing twice with chilled Dulbecco’s PBS supplemented with calcium chloride and magnesium chloride, the resulting cells were treated with trypsin-EDTA solution (Sigma-Aldrich) to recover melanoma cells. After centrifugation at 1000× *g* rpm for 3 min, the cell pellet recovered was dissolved in 100 μL of 1 M NaOH solution (containing 10% DMSO) at 55 °C for 1 h. The melanin content was determined by the absorbance at 405 nm using a microplate reader according to the calibration curve obtained from melanin (Sigma-Aldrich) standards, and melanin synthesis inhibitory activities were expressed as the percentage inhibition of cellular melanin synthesis in the above assay; α-arbutin was used as positive control, while DMSO only was used as negative control.

The in vitro inhibitory effects of SPs and their glycosides on elastase activity (known as anti-wrinkle bioactivities) were assessed using porcine pancreas elastase (Sigma-Aldrich). Its synthetic substrate AAAPVN (Sigma-Aldrich) was dissolved in phosphate buffer (pH 8.0, 200 mM). In each well of a 96-well microplate, the above elastase (20 μL) was mixed with the SPs and their glycosides in the phosphate buffer (150 μL) and then pre-incubated at 25 °C for 15 min prior to substrate AAAPVN (30 μL) supplementation. After 20 min reaction, the absorbance was measured at 402 nm using a microplate reader. The in vitro anti-wrinkle effects through the elastase inhibitory activities were expressed as IC_50_ values (as a concentration of an enzyme inhibitor that caused 50% elastase activity inhibition) by interpolation of the dose–response curves; oleanolic acid (Sigma-Aldrich) was used as positive control, and DMSO only was used as negative control.

### 2.6. Statistical Analyses

All results are expressed as mean ± standard deviations (SD) from at least three independent experiments. The differences between the mean values of the control and the exposed groups were analyzed using one-way analysis of variance (ANOVA) followed by Tukey’s multiple comparisons for comparing means between groups. Differences between means at *p* < 0.01 and *p* < 0.05 (paired or unpaired, two-tailed) were considered significant. 

## 3. Results and Discussion

From the degenerate PCR screening onto the fosmid libraries of an istamycin aminoglycoside-producing *S. tenjimariensis* ATCC 31603, a total of four positive clones were selected then thoroughly sequenced: two were found to be involved in the istamycin aminoglycoside biosynthesis by sequence alignment using BLAST online search tool (http://www.ncbi.nih.gov/BLAST; accessed on 1 July 2022); the remaining two surely encode family I GT; however; they are supposed to act on different kinds of aglycones. Hence, an orf44 (designated with NdeI and XhoI cut) was selected and amplified by PCR, and the amplified ORF of interest (annotated as *stSPGT*) was 1176 base pairs in length, which encodes a predicted 41.78-kDa protein (deposited as GenBank Accession No. MT770755). In silico BLAST analysis of the deduced protein revealed dominant homology with GTs originating from the soil dwelling Actinomycete (including *Micromonospora*, *Streptomyces*, *Actinoplanes* and *Frankia* species). In addition, the presence of a significant conserved motif (as nucleotide-sugar donor binding domain) in the *C*-terminal region indicates that the designated orf44 product StSPGT is a member of the family I GTs.

### 3.1. Enzymatic Biosynthesis of Simple Phenolic Glycosides Using Recombinant StSPGT

**Figure 2:**
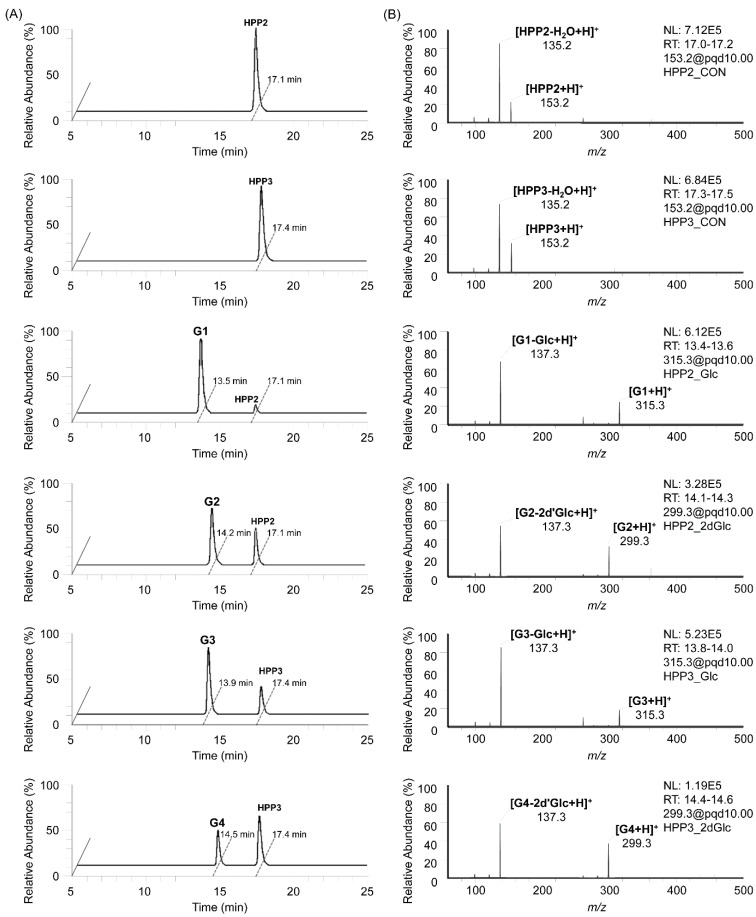
HPLC-MS/MS data of in vitro StSPGT enzymatic reactions. (**A**) HPLC-MS/MS traces of StSPGT’s glycosyl-transferring reactions with both hydroxyphenyl-2-propanol (HPP2) and hydroxyphenyl-3-propanol (HPP3) (as glycosyl acceptors) and two nucleotide sugars, uridine diphosphate-glucose (UDP-Glc) and thymidine diphosphate-2′-deoxyglucose (TDP-2′dGlc). (**B**) Tandem MS spectra of four kinds of glycoside products obtained.

The in vitro enzymatic formation of the corresponding SP glycosides from SP aglycones, together with glycosyl donors, was traced using HPLC-MS/MS analyses, and their MS fragmentation patterns (pairing of mass transitions specific to glycone molecular ion and its dominant aglycone ion corresponding to the loss of a glycosyl moiety) characteristic of the SP glycosides clearly indicated the attachment of glycosyl moieties onto the SP glycosyl acceptors, representing them as a series of SP-O-glycosides (Figure 2); on the HPLC-MS/MS chromatograms obtained from the StSPGT reactants in which UDP-Glc and TDP-2′dGlc were supplemented as the glycosyl donor towards two SP acceptor substrates (HPP2 and HPP3), a total four peaks of glycosylated products were separately traced, thus possibly indicating the substrate-flexible attachment of glycosyl moieties onto both SP aglycones. Further in vitro enzymatic reactions were performed, using two additional common nucleotide-activated sugars (i.e., UDP-GlcA and UDP-GlcNAc) as glycosyl donor substrates of StSPGT to examine whether varied glycosylation occurs or not. However, throughout the overnight reaction, we were not able to trace any targeted SP glycosides as products in the reactant, thus indicating the StSPGT’s limited substrate specificity for the attachment of both Glc and 2′dGlc moieties onto the SP acceptor substrate. The ratios of corresponding SP glycoside to SP aglycone in the reactants were averaged to 97% glycoside **G1** (retention time at 13.5 min) from HPP2 with UDP-Glc, 62% glycoside **G2** (retention time at 14.2 min) from HPP2 with TDP-2′dGlc, 71% glycoside **G3** (retention time at 13.9 min) from HPP3 with UDP-Glc and 44% glycoside **G4** (retention time at 14.5 min) from HPP3 with TDP-2′dGlc (Figure 2A). Three different sets of mass transitions specific to both SPs and their corresponding glycosides were certainly acquired to detect the transition of the protonated precursor ion to the dominant product ion (i.e., 153.2 [M+H]^+^ > 135.2 [M-H_2_O+H]^+^ as a dehydrated product ion equivalent for both HPP2 and HPP3 aglycones; 315.3 > 137.3 [M-Glc+H]^+^ as an aglycone product ion equivalent for glycosides G1 and G3; 299.3 > 137.3 [M-2′dGlc+H]^+^ as an aglycone product ion equivalent for both glycosides G2 and G4) (Figure 2B). Indeed, the recombinant StSPGT seems to recruit HPP2 as a favorable acceptor substrate prior to HPP3, thus revealing its catalytic preference (together with acceptor promiscuity) for the transfer of a glycosyl moiety (glucose or 2′-deoxyglucose) towards SPs. The reverse, namely with respect to the glycosyl donors, also represents that the enzyme is likely to prefer UDP-Glc to TDP-2′dGlc; 97 and 71% conversions from UDP-Glc-supplemented reactions vs. 62 and 44% from TDP-2′dGlc-supplemented ones.

### 3.2. Structural Elucidation of New Simple Phenolic Glycosides

**Figure 3:**
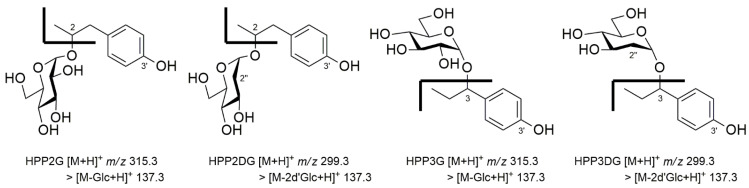
Chemical features of four SP glycosides obtained from StSPGT enzymatic reactions and their MS fragmentation patterns.

In order to obtain and purify enough quantities of SP glycosides for the complete structural elucidation of the glycosyl decoration position onto these glycoside products and the anomeric configuration of attached glycosyl moieties, a normal-phase silica cartridge built in the CombiFlash Rf MPLC system (Teledyne ISCO, Lincoln, NE, USA) was employed. After multiple scale-up reactions followed by MPLC chromatographic separation, a total of four different SP glycosides were obtained as white powder: glycoside **G1** (9.7 mg), glycoside **G2** (6.3 mg), glycoside **G3** (6.8 mg) and glycoside **G4** (3.9 mg). Both HR-MS and NMR instrumental analyses were further performed for the structural elucidation of these SP glycosides, and their structural features were finally described (see Supplementary Tables S1–S4): (i) The ^13^C-NMR spectra of glycosides displayed nine aglycone carbon signals and six carbon signals (as glycosyl group) clearly, and the appearance of proton peaks representing hexosyl moiety within the range of *δ*_H_ 2.11 to 5.05 confirmed that each glycoside contains a glycosyl moiety attached to HPP2 or HPP3 aglycone. (ii) In the ^1^H-NMR spectra, all the glycoside products (**G1** to **G4**) displayed common anomeric proton signals between *δ*_H_ 4.95 to 5.05 (equivalent to the equatorial resonance instead of up fielded-axial one), and the typical observation of noticeable up-field ^13^C glycosylation shifts at each C2-/C3-carbon revealed that a glycosyl group was attached to a free hydroxyl function of the propanoyl moiety, instead being attached to an C3′-hydroxyl function adjacent to phenyl one. (iii) A small coupling constant (J) between 2.6 to 2.9 Hz found at the anomeric proton of all glycosides clearly indicated the introduction of each glycosyl moiety with an α-type glycosidic bond. (iv) The maintenance of proton signals characteristic of each glycosyl moiety (i.e., additional methylene signal [*δ*_H_ 2.25 and 2.13/*δ*_C_ 38.1] at C2″-position of glycosides **G2** and **G4**) confirmed the glycosyl moieties derived from the glycosyl donors were intactly transferred onto the aglycone without further modification (Supplementary Tables S1–S4). Therefore, the enzymatic products **G1** to **G4** were unequivocally assigned as hydroxyphenyl-2-propanoyl-*O*-α-glucoside (HPP2G), hydroxyphenyl-2-propanoyl-*O*-α-2″-deoxyglucoside (HPP2DG), hydroxyphenyl-3-propanoyl-*O*-α-glucoside (HPP3G) and hydroxyphenyl-3-propanoyl-*O*-α-2″-deoxyglucoside (HPP3DG) respectively, and all were even previously unprecedented SP glycosides (Figure 3). Accordingly, the recombinant StSPGT has been shown to attach a series of glycosyl donors with strict regiospecificity at the C2- or C3-hydroxy function present at the propanoyl moiety onto the designated SP aglycone.

### 3.3. Kinetic Features of Recombinant StSPGT

The optimum pH and temperature for the glycosyl-transferring reaction of recombinant StSPGT were investigated using HPP2 and UDP-Glc as an acceptor and a donor substrate, respectively. The enzyme is likely to be stable within the pH range of 5.5 to 8.0 (optimum set at pH 7.4), and the maximum activity was found at 30 °C (data not shown). To learn more details about the enzymatic function of a recombinant StSPGT, we investigated and determined its bio-catalytic properties using two nucleotide sugars as the glycosyl donor substrates; the production velocities of the corresponding glycosides were plotted against the varied glycosyl donor concentrations, instead of with fixed acceptor concentrations. The *K*_m_ and *k*_cat_ values for UDP-Glc donor substrate of the enzyme was averaged to be 0.16 mM and 19.41 min^−1^, respectively, for HPP2 and 0.22 mM and 16.87 min^−1^, respectively, for HPP3 (Table 1 and Supplementary Figure S1). The *K*_m_ values of recombinant StSPGT for the Glc-transferring activity onto HPP2 and HPP3 were both rigid within the range of 0.16 to 0.22 mM and comparable; however, they were noticeably lower than those determined for another 2′dGlc-transferring substrate. Indeed, the enzyme displayed the lowest *K*_m_ value (0.16 mM) for the glucosylation of HPP2 compared to those for HPP3 (0.22 mM), displaying StSPGT’s preference for HPP2 as the acceptor substrate over HPP3. These apparent *K*_m_ values appear to be comparable to those reported for other microbial- or plant-origin simple phenolic glycosyltransferases [1,16,22,28]. Depending upon the donor substrates, several fold differences were found in the *K*_m_ values; 0.16 and 0.22 mM when a Glc moiety was incorporated into each aglycone vs. 0.41 and 0.50 mM when a 2′dGlc-moiety was. In particular, the glycosyl donor supplement, especially UDP-Glc onto StSPGT’s enzymatic reactions with HPP2 aglycone, gave the highest turnover number (*k*_cat_ = 19.41 min^−1^) (Table 1 and Supplementary Figure S1). However, the incorporation of another glycosyl donor (TDP-2′dGlc) into the reactions resulted in more remarkably increased *K*_m_ and decreased *k*_cat_ values, hence clearly demonstrating UDP-Glc as a favorable glycosyl donor substrate. The thorough kinetic analyses revealed that the incorporation of both Glc and 2′dGlc moieties onto SP acceptors can be bio-catalyzed by StSPGT, but the latter had catalytic efficiencies only one-fourth as high as for the UDP-Glc donor, as judged by *k*_cat_/*K*_m_ values (Table 1). Indeed, the *k*_cat_ values obtained from UDP-Glc towards HPP2 (19.41 min^−1^) and HPP3 (16.87 min^−1^) were considerably high, thereby indicating StSPGT’s higher catalytic efficiency (107.83 min^−1^ mM^−1^ for HPP2 and 76.68 min^−1^ mM^−1^ for HPP3) compared with that derived from another glycosyl donor; it is obvious that StSPGT can utilize two different nucleotide sugars as glycosyl donors as well as both SP aglycones as glycosyl acceptors, undoubtedly indicating its promiscuity towards pairs of substrates. Based on the kinetic data obtained (Table 1) and the structural features of the SP aglycones (Figure 1 and Figure 3), we were able to speculate on the relationship between the substrate structure and the enzyme’s catalytic activity. This region-specific StSPGT can distinguish, albeit with different substrate spectra and catalytic efficiencies, the positional difference of a hydroxyl function (as C2- or C3-hydroxy) attached onto the propanoyl moiety of each HPP2 and HPP3, and therefore the three-dimensional structure of StSPGT might provide valuable insights into the plausible protein–ligand (or catalytic site-ligand) interactions.

### 3.4. Antioxidant, Anti-Melanogenic and Anti-Wrinkle Properties

**Figure 4:**
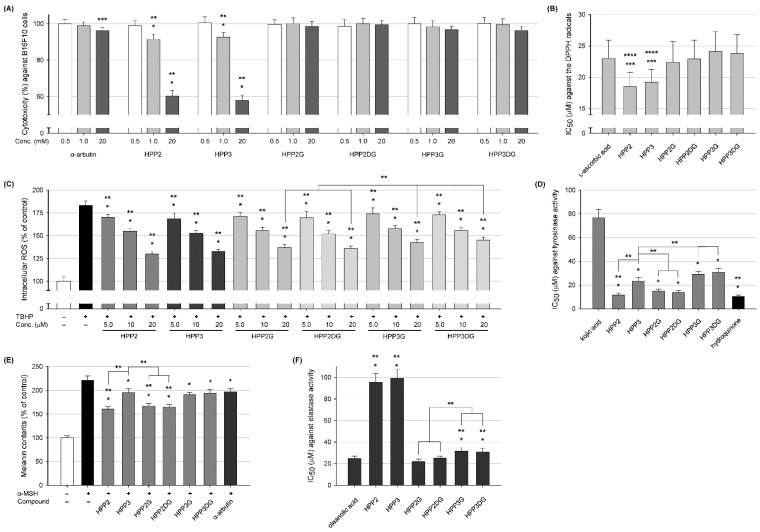
Biological activities of SP aglycones and their glycosides. (**A**) cytotoxicity against B16F10 murine melanoma cells (α-arbutin as a reference), (**B**) in vitro DPPH radical scavenging antioxidant activity represented as IC_50_ values (L-ascorbic acid as a positive control), (**C**) in vivo ROS scavenging antioxidant activity against *tert*-butyl hydroperoxide (TBHP)-induced B16F10 cells, (**D**) in vitro tyrosinase inhibitory activity represented as IC_50_ values (kojic acid as a positive control, and hydroquinone as a comparative one), (**E**) inhibitory activity against the intracellular melanin biosynthesis in B16F10 cells (α-arbutin as a positive control) at fixed 20 μM treatments, and (**F**) in vitro elastase inhibitory activity (oleanolic acid as a positive control). HPP2: hydroxyphenyl-2-propanol; HPP3: hydroxyphenyl-3-propanol; HPP2G: hydroxyphenyl-2-propanoyl-*O*-α-glucoside; HPP2DG: hydroxyphenyl-2-propanoyl-*O*-α-2″-deoxyglucoside; HPP3G: hydroxyphenyl-3-propanoyl-*O*-α-glucoside; HPP3DG: hydroxyphenyl-3-propanoyl-*O*-α-2″-deoxyglucoside. Results (* *p* < 0.01 vs. control; ** *p* < 0.01 vs. treatments; *** *p* < 0.05 vs. control; **** *p* < 0.05 vs. treatments) were considered significant.

After elucidating the structural features of the SP glycosides obtained, their biological activities (including cytotoxicity, antioxidant, anti-melanogenic and anti-aging activities) were evaluated by comparison with those of both parental aglycones and the positive controls (i.e., L-ascorbic acid, kojic acid, hydroquinone, α-arbutin and oleanolic acid). The treatment concentrations of SP aglycones and their glycosides for the bioassays were selected in advance by monitoring their cytotoxicity against the B16F10 murine melanoma cell line using α-arbutin as the reference. Cell viability was measured after 48 h treatments with different concentrations (0.5, 1.0, 5.0, 10 and 20 mM) and is summarized in Figure 4A (overall depicted in Supplementary Figure S2); a reference compound, α-arbutin, displays a slight change (less than 5% of the control) of cell viability only at the highest 20 mM (equivalent to 0.6% content) concentration. In 2015, the European Scientific Committee on Consumer Safety issued a report on the safety of α-arbutin; its cosmetic use is safe for consumers, with concentrations up to 0.5% in skin-care products [15]. Previous publication has also revealed that α-arbutin has no cytotoxicity for the B16F10 cells if the concentrations are less than 5.0 mM concentrations [29], thus accordant with the observations herein. Quite similar to the result of α-arbutin, all the glycosides also present a very high percentage of viability with increasing concentrations up to the maximum tested, whereas both aglycones exhibit significantly decreased cell viabilities at concentrations lower than 1.0 mM in B16F10 cells, thus suggesting the SP glycosylation seems to considerably alleviate the cytotoxicity of parental SP aglycones. Accordingly, the further bioassays were conducted for all test samples at concentrations less than the 1.0 mM level.

The in vitro antioxidant activity of SP aglycones and their corresponding glycosides was evaluated through their DPPH-driven radical scavenging abilities in comparison with a well-known antioxidant, L-ascorbic acid. The IC_50_ values obtained from a total of four kinds of glycosides were examined to be within 22.4 to 24.1 μM, comparable to that (23.0 μM) obtained from the positive control L-ascorbic acid. In the latest publication, the IC_50_ value of L-ascorbic acid was evaluated at 22.0 μM through its DPPH radical scavenging abilities, very similar to the foregoing results [30]. On the other hand, two SP aglycones provided significantly lower IC_50_ values (18.5 μM for HPP2 and 19.3 μM for HPP3, respectively) than the positive control, indicating a substantial radical scavenging capacity (Figure 4B). Therefore, compared with L-ascorbic acid, the series of SP glycosides obtained still showed comparable DPPH radical scavenging ability, hence implying a considerable antioxidant activity remained in the SP glycosides of interest. Although the mean IC_50_ values of HPP2G and HPP2DG appear to be a little lower than those of HPP3G and HPP3DG, respectively, there was, however, no statistically significant difference found within (also between) these glycoside groups. Next, we implemented a DCFDA cell-based ROS detection assay [31] to determine whether the SP aglycones and their glycoside analogs function in vivo as antioxidants against intracellular ROS synthesis in the above-described B16F10 cells or not. The TBHP-induced cells were further incubated with different concentrations (5.0, 10 and 20 μM) of SPs and their glycosides for varied time periods (10, 30 min and 1 h). As shown in Figure 4C, the TBHP-exposure induced an approximately 1.8-fold increase in the intracellular ROS level compared with the basal ROS level.

As expected, treatments of both SP aglycones and their glycosides were likely to attenuate the TBHP-mediated ROS level evenly in a concentration-dependent manner. In addition, their treatments at fixed 20 μM with extended time periods (up to 1 h) also led to the reduced intracellular ROS levels in a time-dependent manner (Supplementary Figure S3). These results clearly demonstrated that the SPs and their glycosides possess antioxidant activity, in a time- and concentration-dependent manner, by scavenging DPPH radicals as well as the intracellular ROS. Interestingly, even though the results obtained from cell-based ROS assay are almost consistent with those from the DPPH assay, HPP2-type glycosides appeared to act as more potent ROS-scavenging antioxidants than HPP3-type glycosides (Figure 4C); the maximal ROS-scavenging activities of HPP2G and HPP2DG (52.0 and 54.3%, respectively) were notably higher than those of HPP3G and HPP3DG (48.9 and 45.7%, respectively). Although their ROS-scavenging activities were entirely inferior to the parental aglycones, the positional glycosylation at C2- or C3-hydroxyl function onto their propanoyl moieties resulted in the distinctive ROS-scavenging results. After all, the conformational changes caused by the glycosyl attachment (or shielding) of the free C3-hydroxy group (not at C2-position) tend to weaken ROS-scavenging antioxidant potentials.

The comparative tyrosinase inhibitory activities of SPs and SP glycosides were evaluated and expressed as IC_50_ values; kojic acid (one of the famous skin-whitening agents) was used as a positive control and hydroquinone (also known as a skin-depigmenting agent with the strongest tyrosinase inhibitory activity) was used as a comparison for in vitro tyrosinase inhibition [32]. As illustrated in Figure 4D, the IC_50_ value of positive control kojic acid was evaluated as 76.5 μM; kojic acid has previously been proven to have stronger tyrosinase inhibition ability than many other commercial inhibitors (including α-arbutin and L-ascorbic acid). Among the compounds tested, the most potent inhibition was shown by hydroquinone (10.3 μM), followed by HPP2, HPP2 glycosides, HPP3 and HPP3 glycosides in that order. Indeed, the IC_50_ values of HPP2 and HPP3 aglycone were determined to be 11.6 and 23.4 μM, respectively, indicating about 6.6-fold and 3.3-fold higher tyrosinase inhibitory effects, respectively, than kojic acid. In particular, an HPP2 aglycone was the most potent tyrosinase inhibitor, of which IC_50_ value seems to be comparable to that of hydroquinone. Moreover, different form the above-described antioxidant results, where both SP aglycones are categorized into the same group, the tyrosinase inhibitory activities of both SP aglycones were markedly different. In case of SP glycosides, a similar inhibitory tendency described just above was still verified; IC_50_ values of HPP2G and HPP2DG were averaged to be 14.8 and 13.8 μM, respectively, while those of HPP3G and HPP3DG were distinctively 29.1 and 30.9 μM, respectively (Figure 4D). It was evident from these results that not only the existence of C2-hydroxy function onto a propanoyl moiety of the SP aglycone but also the glycosyl decoration at the C2-position are likely to be responsible for an enhanced tyrosinase inhibitory activity of the corresponding compounds. However, there was no difference in tyrosinase inhibitory activities depending on the type of glycoside (Glc or 2d′Glc). Therefore, it was certainly determined that the glycosyl attachment at the SP’s C2-hydroxyl group does not attenuate the parental activity appreciably, thus suggesting both HPP2 aglycone and the corresponding glycosides as promising tyrosinase inhibitors, applicable as skin-depigmenting dermatological agents. Further studies on molecular docking and dynamic simulation are requisite for understanding the physical and biochemical bases of HPP2 glycosides towards tyrosinase inhibition [33]. Melanin biosynthesis is a stress response of melanocytes, which can cause melanosis, such as freckles, brown spots, melanoma, senile plaques and even melanocytoma [15,30]. To confirm whether the in vitro tyrosinase inhibition found in the preceding experiment was still correlated with the inhibition of the in vivo melanin biosynthesis, we investigated the inhibitory effects of the SPs and their corresponding glycosides on the α-MSH-induced melanin biosynthesis in B16F10 cells. Treatment with α-MSH induced an approximately 2.2-fold increase in the intracellular melanin content (Figure 4E). When it came to the positive control α-arbutin, inhibition activities of α-MSH-induced intracellular melanin biosynthesis turned out to be about 20.3%, and HPP2 aglycone exhibited the highest inhibitory potential with 50.3%. Additionally, SP glycosides all were in-between, showing inhibitory activities within a range between 22.4 and 46.5%, and the HPP3 aglycone was ranked the lowest (21.7%) (Figure 4E). Furthermore, the α-MSH-induced melanogeneses were all reversed with concentration-dependent manner (Supplementary Figure S4). These findings were almost in harmony with the preceding tyrosinase inhibitory results, thus demonstrating that HPP2 and their glycosides (i.e., HPP2G and HPP2DG), albeit with their varied activities, are not only able to inhibit tyrosinase activity in vitro but also undoubtedly suppressed in vivo melanogenesis.

Elastase, a protease, is responsible for the breakdown of elastin in the extracellular matrix (ECM). Elastase can break down elastin along with other substrates (i.e., collagen and fibronectin). In order to decelerate the tangible impacts of skin-aging (i.e., wrinkles, freckles and sagging), the degradation of elastin substrate derived from the elastase activity should be interrupted, thereby terminating the depletion of skin elasticity. Herein, the positive control oleanolic acid displayed an IC_50_ value of 24.9 μM (Figure 4F). A previous study has also shown that the IC_50_ value of oleanolic acid (still represented as a positive control) for the elastase inhibitory experiments was 11.7 μg/mL, equivalent to a molar concentration of 25.7 μM [34]. Hence, it was found that this converted molar concentration was quite resemblant to ours (24.9 μM). In addition, the IC_50_ values of both SP aglycones against the porcine elastase were averaged to be 95.3 and 99.1 μM, respectively, whereas those of HPP3G and HPP3DG were 31.5 and 30.7 μM, respectively, hence indicating that the inhibitory activities of these samples against elastase appeared to be lower than that of oleanolic acid. Contrary to this, a series of HPP2 glycosides are likely to exert the best inhibitory activity against elastase with IC_50_ values (22.1 and 25.3 μM, respectively) lower than (or comparable to) that of the positive control oleanolic acid (Figure 4F), thus clearly demonstrating these HPP2 glycosides with enhanced anti-melanogenic and antioxidant activities to be prospective anti-wrinkle cosmetic hits.

## 4. Conclusions

From the degenerate PCR screening onto the fosmid libraries of *S. tenjimariensis* ATCC 31603, followed by in silico analyses, we for the first time isolated StSPGT as a member of family I GTs. Its enzymatic function was proven as an SP glycosyltransferase, which utilizes nucleotide-activated sugar as the glycosyl donor for the attachment of this sugar moiety on the acceptor substrate SP aglycone. The presence of a total of four products derived from the recombinant GT reaction demonstrated the StSPGT’s limited substrate specificity for the attachment of both Glc and 2′dGlc moieties onto the SP acceptor substrate (Figure 2). The chromatographic separation of product glycosides followed by the instrumental analyses unveiled that a glycosyl group was attached to a free hydroxyl function of the propanoyl moiety of the SP aglycones with strict regiospecificity, instead being attached to a C′4-hydroxyl function adjacent to a phenyl one. Furthermore, the anomeric configuration of the glycosyl moiety attached was proven as the α-configuration. Therefore, these a series of the previously unprecedented SP glycosides were assigned as hydroxyphenyl-2-propanoyl-*O*-α-glucoside (HPP2G), hydroxyphenyl-2-propanoyl-*O*-α-2″-deoxyglucoside (HPP2DG), hydroxyphenyl-3-propanoyl-*O*-α-glucoside (HPP3G) and hydroxyphenyl-3-propanoyl-*O*-α-2″-deoxyglucoside (HPP3DG) (Figure 3). Different from the previous publications dealing with plant- and/or microbe-origin UGT-aided β-anomeric glycosylation, the enzyme StSPGT was able to transfer two glycosyl moieties (i.e., Glc and 2′dGlc), albeit with contrasting biocatalytic properties towards the glycosyl donors, onto both SP aglycones (i.e., HPP2 and HPP3) with an α-glycosidic linkage. Indeed, the enzyme possesses a limited glycosyl donor substrate flexibility and can be utilized as a regio- and stereo-selective biocatalyst. In addition, these findings also provided meaningful insight into the capability of the microbial UGT for its regio-specific manufacturing of a target glycoside in accordance with a particular glycosyl-transferring donor substrate add-on. As proof for the above-described biocatalytic properties of the recombinant StSPGT, thorough enzymatic kinetic studies were conducted and then surely confirmed that this regio-specific StSPGT can distinguish, albeit with different substrate spectra and catalytic efficiencies, the positional difference of a hydroxyl function (as C2- or C3-hydroxy) attached onto the propanoyl moiety of each HPP2 and HPP3. Moreover, this recombinant StSPGT prefers UDP-Glc and HPP2 as a favorable glycosyl donor and acceptor substrate, respectively (Table 1). The biological activities of a total of six kinds of compounds (including two SP aglycones and four different SP glycosides) were evaluated (Figure 4) and then summarized as follows: (a) According to the cell viability test, SP aglycones exhibits significantly decreased cell viabilities at concentrations as lower than 1 mM in B16F10 cells, hence further bioassays were conducted for all test samples at concentrations less than 1 mM level. (b) The paired in vitro and in vivo antioxidant assays indicated that the SPs and their glycosides possess antioxidant activity, in a time- and concentration-dependent manner, by scavenging DPPH radicals and also the intracellular ROS. Interestingly, HPP2-type glycosides appeared to act as more potent ROS-scavenging antioxidants than HPP3-type glycosides. (c) The existence of C2-hydroxy function onto a propanoyl moiety of the SP aglycone as well as the glycosyl decoration at the C2-position are likely to be responsible for an enhanced tyrosinase inhibitory activity of the corresponding compounds. Therefore, HPP2 and their glycosides (i.e., HPP2G and HPP2DG) are not only able to inhibit tyrosinase activity in vitro but also undoubtedly suppress in vivo melanogenesis. (d) HPP2 glycosides are likely to exert the best inhibitory activity against elastase compared with the positive control oleanolic acid, thus clearly demonstrating these HPP2 glycosides with enhanced anti-melanogenic and antioxidant activities as prospective anti-wrinkle dermatological preparations. Further studies on both the GT kinetics of StSPGT on other related substrates and its introduction into the microbial cell factory for the in vivo production of desirable SP glycosides might be carried out [35], and the resulting biosynthetic new glycosides obtained from their biosynthetic platforms might also contribute to the glyco-expansibility of SPs with updated industrial potentials.

## 5. Patents

Korea University has registered a Korean patent (10-1884382) covering part of the work presented in this article, listing J.W.P. as the inventor.

## Figures and Tables

**Table 1:** Kinetic parameters for a recombinant StSPGT with both SP aglycone acceptors (HPP2 and HPP3) and two glycosyl donors (uridine diphosphate-glucose [UDP-Glc] and thymidine diphosphate-2′-deoxy-glucose [TDP-2′dGlc]).

Acceptors	Donors	*K*_m_ (mM)	*k*_cat_ (min^−1^)	*k*_cat_/*K*_m_ (mM^−1^ min^−1^)
HPP2	UDP-Glc	0.16 ± 0.03	19.41 ± 3.22	107.83
	TDP-2d′Glc	0.41 ± 0.07	12.93 ± 2.18	31.54
HPP3	UDP-Glc	0.22 ± 0.06	16.87 ± 2.98	76.68
	TDP-2d′Glc	0.50 ± 0.09	11.19 ± 2.23	22.38

All kinetic data represent mean ± standard deviation (*n* = 3), derived from the Michaelis–Menten equation.

## Data Availability

The data is contained within this article and Supplementary Files.

## Supplementary Materials

The following supporting information can be downloaded at: https://www.mdpi.com/article/10.3390/antiox11071396/s1, Supplementary Methods; Table S1: ^1^H- and ^13^C-NMR data (500 MHz; DMSO-*d_6_*) of glycoside **G1** (hydroxyphenyl-2-propanoyl-O-α-glucoside; Table S2: ^1^H- and ^13^C-NMR data (500 MHz; DMSO-*d_6_*) of glycoside **G2** (hydroxyphenyl-2-propanoyl-O-α-2″-deoxyglucoside; Table S3. ^1^H- and ^13^C-NMR data (500 MHz; DMSO-*d_6_*) of glycoside **G3** (hydroxyphenyl-3-propanoyl-O-α-glucoside; Table S4. ^1^H- and ^13^C-NMR data (500 MHz; DMSO-*d_6_*) of glycoside **G4** (hydroxyphenyl-3-propanoyl-O-α-2″-deoxyglucoside; Figure S1. Michaelis-Menten kinetics for recombinant StSPGT-catalyzed production of SP glycosides using the fixed glycosyl acceptor concentration with the varied glycosyl donor concentrations; Figure S2. Cytotoxicity of SP aglycones and their biosynthetic glycosides against B16F10 murine melanoma cells with varied concentrations (0.5, 1.0, 5.0, 10 and 20 mM). α-arbutin was utilized as a positive control; Figure S3. The in vivo ROS scavenging antioxidant activity of SP aglycones and their biosynthetic glycosides against tert-butyl hydroperoxide (TBHP)-induced B16F10 cells at fixed 20 μM treatments with extended time intervals (10, 30 min and 1 h); Figure S4. The inhibitory activity of SP aglycones and their biosynthetic glycosides against the intracellular melanin biosynthesis in B16F10 cells with varied concentrations (5.0, 10 and 20 μM). α-arbutin was utilized as a positive control.
