# Supplementary material for: Enzymatic Biosynthesis of Simple Phenolic Glycosides as Potential Anti-Melanogenic Antioxidants
Source: Antioxidants (Basel). 2022 Jul 19;11(7):1396. doi: 10.3390/antiox11071396 (PMC9312196; doi:10.3390/antiox11071396)
Supplement: Supplementary file 1 [file antioxidants-11-01396-s001.zip › antioxidants-1825413-supplementary.pdf]

SUPPLEMENTARY MATERIALS

Enzymatic Biosynthesis of Simple Phenolic Glycosides As Potential Anti-melanogenic Antioxidants

Hogwuan Jung [1,4], JaeWook Oh [1,4], Younghae Kwon [1], Woongshin Kang [1], Minsuk Seo [1], Yurin Seol [1] and Je Won Park [2,3,*]

[1] Transdisciplinary Major in Learning Health Systems, Department of Integrated Biomedical and Life Sciences, Korea University, Seoul 02841

[2] School of Biosystems and Biomedical Sciences, Korea University, Seoul 02841

[3] Department of Integrated Biomedical and Life Sciences, Korea University, Seoul 02841, Republic of Korea

[4] These authors contributed equally

* Correspondence: jewonpark@korea.ac.kr; Tel.: +82-2-3290-5645

Supplementary Methods

Chemicals and Reagents

Thymidine diphosphate-2'-deoxy-glucose (TDP-2'dGlc) was from GeneChem (Daejeon, Republic of Korea). Isopropyl β-D-1-thiogalactopyranoside (IPTG), kanamycin, imidazole, uridine diphosphate-glucose (UDP-Glc), UDP-glucuronic acid (UDP-GlcA), UDP-N-acetyl glucosamine (UDP-GlcNAc), formic acid, *tert*-butyl hydroperoxide (TBHP), L-ascorbic acid, tyrosinase, kojic acid, α-arbutin, hydroquinone, α-melanocyte stimulating hormone, trypsin-EDTA solution, melanin, 3-(4,5-dimethylthiazol-2-yl)-2,5-diphenyltetrazolium bromide (MTT), elastase (from porcine pancreas), *N*-succinyl-(L-alanine-alanine-alanine)-*p*-nitroanilide (AAAPVN) and oleanolic acid were obtained all from Sigma-Aldrich (St. Louis, MO, USA). The mouse melanoma cell line B16F10 was purchased from the American Type Culture Collection (ATCC, Manassas, VA, USA). HPLC-grade solvents and water were purchased from JT Baker (Center Valley, PA, USA), and others were of reagent grade.

Isolation and Heterologous Expression of Recombinant StSPGT in E. coli

Fosmid genomic library of *S. tenjimariensis* ATCC 31603 was constructed in CopyControl pCC1FOS according to the manufacturer's instructions (Epicentre Technologies, Madison, WI, USA). These fosmid clones were separately incubated overnight at 37°C in individual wells of 96-well microtiter plates, which contained 0.6 mL of LB broth supplemented with chloramphenicol. From the cell pellets harvested by centrifugation, the fosmid DNAs were isolated by using a BACMAX96 DNA purification kit (Epicentre Technologies), and then used as template for degenerate PCR; forward (5′-ATCCACGCGCACGACTTCCGGATG-3′) and reverse (5′-TTCGGCCTGCGCCTCCCACGTCCA-3′) primer pair was designated. Through the degenerate PCR screening for the GT-encoding gene onto the fosmid libraries of an istamycin aminoglycoside-producing *S. tenjimariensis* ATCC 31603, an orf44 (designated with NdeI and XhoI cut) was selected and amplified by PCR. The amplified PCR products were subcloned into

the pGEM T-Easy vector (Promega, Madison, WI, USA) and transformed into *E. coli* XL1-Blue strain (Stratagene, La Jolla, CA, USA). The resultant plasmid obtained from was sequenced; the amplified ORF of interest (annotated as StSPGT) was deposited as GenBank Accession No. MT770755.

The *Nde*I/*Xho*I-digested fragment excised from the aforementioned pGEM T-Easy vector was cloned into the same sites of the expression vector pET-28a(+) (Novagen, San Diego, CA, USA) and expressed as an *N*-terminal fusion protein with an His_6 tag in *E. coli* BL21(DE3) strain (selected with 50 mg/mL kanamycin). After induction using 0.5 mM IPTG (Sigma-Aldrich) followed by an overnight incubation at 22°C, the cells were harvested and then disrupted by ultrasonication. The His_6-tagged protein from the lysate was purified with TALON-metal affinity resin (Clontech, Mountain View, CA, USA) equilibrated with 50 mM phosphate buffer (0.3 M NaCl, 20 mM imidazole) and incubated at 4°C for 1 hr. After 5 min of refrigerated centrifuge at 2000 rpm, the resin was introduced into a disposable column and washed with phosphate buffer containing 20 mM imidazole, equivalent to 8 times the amount of the resin. Finally, recombinant StSPGT bound to the above resin was purified with 3 mL of phosphate buffer containing 150 mM imidazole. The protein concentration was determined according to the Bradford method and the purified StSPGT was stored at −20°C prior to enzymatic reactions.

In vitro Enzymatic GT Reactions and Its Kinetic Studies

After dissolving two SP acceptor substrates, including HPP2 and HPP3, in methanol at 100 mM, we diluted them with a reaction buffer solution (50 mM phosphate buffer, 10 mM magnesium chloride and 1 mg/mL bovine serum albumin; pH 7.4) to the final concentration of 1 mM. Then we added 30 μM StSPGT glycosyltransferase along with four different nucleotide-activated glycosyl donors (UDP-Glc, UDP-GlcA, UDP-Gal and TDP-2'dGlc) at 2 mM and subsequently conducted GT reaction at 30°C for 30 min. After reaction, an equal volume of ethyl acetate was immediately added to quench the reaction. The organic solvent layer was evaporated to dryness at 40°C by

vacuum centrifugation (EYELA, Tokyo, Japan), and finally reconstituted in methanol prior to HPLC-tandem mass spectrometric (MS/MS) analysis.

For the determination of the kinetic parameters for each SP acceptor substrate, the concentration of glycosyl donor was fixed at 2 mM, while the concentration of SP was varied from 0.1 to 1.2 mM. To measure the kinetic properties with respect to each glycosyl donor substrate, the concentration of the SP was kept constant at 1.0 mM, while the concentration of glycosyl donor was varied from 0.1 to 1.2 mM. All reactions were quenched with ethyl acetate. After the evaporation of the organic solvent layer to dryness, aliquots of extracts reconstituted in methanol, were subjected to HPLC-MS/MS analyses as described below. Each experiment was performed in triplicate, and the reaction mixture containing boiled StSPGT served as control. The Michaelis-Menten kinetic model was fitted to the data of production rates of the designated glycosides versus the respected substrate concentrations. Equation fitting and corresponding kinetic properties were acquired using the SigmaPlot enzyme kinetic module (Systat Software Inc., ver. 12.0, San Jose, CA, USA). The kinetic parameters which include maximal velocity V_{max}, Michaelis-Menten constant K_m, turnover number k_{cat}, and catalytic efficiency k_{cat}/K_m were measured and represented as mean ± standard deviation (in triplicates) relative to the Michaelis-Menten equation.

Instrumental Analyses

Quantitative analysis of the reaction products was performed by HPLC-MS/MS consisting of a Spectra SYSTEM P1000XR quaternary pump and a Spectra series AS3000 auto-sampler equipped with a 20-µL loop, both of which were obtained from ThermoFinnigan (San Jose, CA, USA). Chromatography was performed with an Acquity CSH C18 reversed-phase column (Waters, Milford, MA, USA; 2.1 × 50 mm; 1.7 µm) at a flow rate of 130 µL/min using an isocratic mobile phase (MeOH:CH3CN:water:formic acid = 10:50:39.9:0.1, v/v/v/v). The column effluent was introduced into the MS/MS (without splitting), which was operated in the positive ion mode.

Acquisition was performed using MS/MS operated in the selective reaction monitoring mode by choosing different sets of mass transitions specific to both SPs and their corresponding glycosides.

To produce sufficient quantities of the SP glycosides for the purpose of elucidating their chemical structure, GT reactants from more than eight scale-up (each 1.5 mL) batches were pooled and extracted as described above. The upper solvent layer obtained was evaporated to dryness, and then reconstituted in 5 mL of the mobile phase utilized for HPLC-MS/MS analyses. The extracts were loaded onto a reversed-phase C_{18} cartridge of the CombiFlash Rf medium-pressure liquid chromatography (MPLC) system (Teledyne ISCO, Lincoln, NE, USA), and the flow rate was set at 7 mL/min. The eluents passing through a UV detector were all automatically fractionated over 40-min running time. The fractions containing the target glycoside with a high purity (> 95%) by the tracing HPLC-MS/MS analyses, were pooled and freeze-dried. Chemical structures of glycosides designated were further confirmed by Varian INOVA 500 nuclear magnetic resonance (NMR, Varian Inc., Palo Alto, CA, USA) spectroscopic analysis together with high resolution (HR) LCT-premier XE MS (Waters, Milford, MA, USA) analysis.

Supplementary Table S1. ^{1}H- and ^{13}C-NMR data (500 MHz; DMSO-d_6) of glycoside **G1** (hydroxyphenyl-2-propanoyl-*O*-α-glucoside; HPP2G; 9.7 mg as a white powder; molecular formula $C_{15}H_{22}O_7$, $[M+H]^+$ *m/z* 315.1436, calculated 315.1438), compared with those of hydroxyphenyl-2-propanol (HPP2) aglycone.

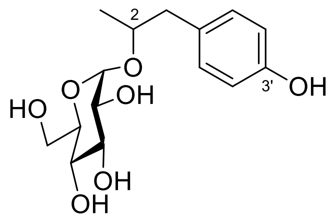

	HPP2G		HPP2	
	^{1}H (ppm)	^{13}C (ppm)	^{1}H (ppm)	^{13}C (ppm)
Aglycone				
1	1.18	21.2	1.17	23.1
2	3.42	60.3	3.78	70.4
3	2.56	44.2	2.56	46.1
	2.81		2.82	
4		130.6		130.7
1'	7.08	128.0	7.10	128.5
2'	6.61	115.7	6.65	116.2
3'		155.3		155.5
4'	6.72	114.7	6.71	114.6
5'	7.13	125.7	7.14	125.7
2-OH			3.55	
3'-OH	5.35		5.34	
Glucose				
1''	5.05 *d* (2.7)	109.4		
2''	3.71 *m*	74.0		
3''	3.49 *dd*	76.6		
4''	3.40 *dd*	71.5		
5''	3.74 *m*	81.4		
6''	3.78 *d*	62.1		
	3.54 *m*			

Coupling constant (*J* in Hz) in parentheses

Supplementary Table S2. ^{1}H- and ^{13}C-NMR data (500 MHz; DMSO-d_6) of glycoside **G2** (hydroxyphenyl-2-propanoyl-*O*-α-2''-deoxyglucoside; HPP2DG; HPP2G; 6.3 mg as a white powder; molecular formula $C_{15}H_{22}O_6$, $[M+H]^+$ *m/z* 299.1487, calculated 299.1489), compared with those of hydroxyphenyl-2-propanol (HPP2) aglycone.

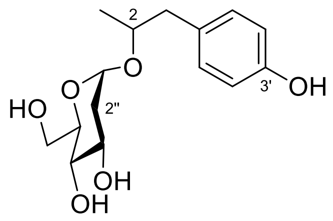

	HPP2DG		HPP2	
	^{1}H (ppm)	^{13}C (ppm)	^{1}H (ppm)	^{13}C (ppm)
Aglycone				
1	1.17	21.3	1.17	23.1
2	3.44	60.1	3.78	70.4
3	2.53	44.2	2.56	46.1
	2.80		2.82	
4		130.6		130.7
1'	7.07	128.1	7.10	128.5
2'	6.60	115.9	6.65	116.2
3'		155.4		155.5
4'	6.70	114.6	6.71	114.6
5'	7.11	125.7	7.14	125.7
2-OH			3.55	
3'-OH	5.34		5.34	
2''-Deoxyglucose				
1''	4.96 *d* (2.7)	105.5		
2''	2.25 *d*	38.1		
	2.13 *m*			
3''	3.41 *dd*	68.9		
4''	3.39 *dd*	71.3		
5''	3.74 *m*	77.6		
6''	3.79 *d*	62.2		
	3.53 *m*			

Coupling constant (*J* in Hz) in parentheses

Supplementary Table S3. ^{1}H- and ^{13}C-NMR data (500 MHz; DMSO-d_6) of glycoside **G3** (hydroxyphenyl-3-propanoyl-*O*-α-glucoside; HPP3G; 6.8 mg as a white powder; molecular formula $C_{15}H_{22}O_7$, $[M+H]^+$ *m/z* 315.1434, calculated 315.1438), compared with those of hydroxyphenyl-3-propanol (HPP3) aglycone.

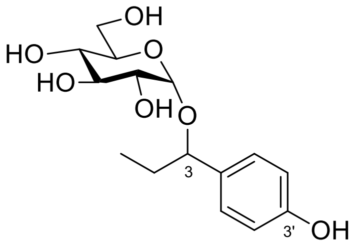

	HPP3G		HPP3	
	^{1}H (ppm)	^{13}C (ppm)	^{1}H (ppm)	^{13}C (ppm)
Aglycone				
1	0.91	10.3	0.94	10.2
2	1.84	30.1	1.84	31.5
	1.64		1.65	
3	4.13	63.3	4.40	76.3
4		134.5		137.1
1'	7.18	126.6	7.19	126.6
2'	6.70	115.0	6.70	115.1
3'		155.8		155.8
4'	6.62	115.7	6.64	116.0
5'	7.10	127.8	7.14	128.3
3-OH			3.62	
3'-OH	5.35		5.35	
Glucose				
1''	5.03 *d* (2.6)	111.6		
2''	3.72 *m*	74.1		
3''	3.49 *dd*	76.7		
4''	3.41 *dd*	71.5		
5''	3.75 *m*	81.5		
6''	3.79 *d*	62.2		
	3.54 *m*			

Coupling constant (*J* in Hz) in parentheses

Supplementary Table S4. ^{1}H- and ^{13}C-NMR data (500 MHz; DMSO-d_6) of glycoside **G4** (hydroxyphenyl-3-propanoyl-*O*-α-2''-deoxyglucoside; HPP3DG; 3.9 mg as a white powder; molecular formula $C_{15}H_{22}O_6$, $[M+H]^+$ *m/z* 299.1486, calculated 299.1489), compared with those of hydroxyphenyl-3-propanol (HPP3) aglycone.

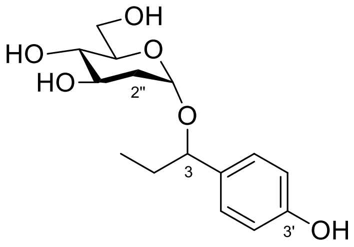

	HPP3DG		HPP3	
	^{1}H (ppm)	^{13}C (ppm)	^{1}H (ppm)	^{13}C (ppm)
Aglycone				
1	0.91	10.1	0.94	10.2
2	1.83	29.9	1.84	31.5
	1.64		1.65	
3	4.12	63.1	4.40	76.3
4		134.6		137.1
1'	7.19	126.6	7.19	126.6
2'	6.70	115.0	6.70	115.1
3'		155.6		155.8
4'	6.61	115.8	6.64	116.0
5'	7.10	128.0	7.14	128.3
3-OH			3.62	
3'-OH	5.33		5.35	
2''-Deoxyglucose				
1''	4.95 *d* (2.8)	104.5		
2''	2.25 *d*	38.1		
	2.13 *m*			
3''	3.41 *dd*	68.9		
4''	3.38 *dd*	71.2		
5''	3.75 *m*	77.5		
6''	3.78 *d*	62.0		
	3.54 *m*			

Coupling constant (*J* in Hz) in parentheses

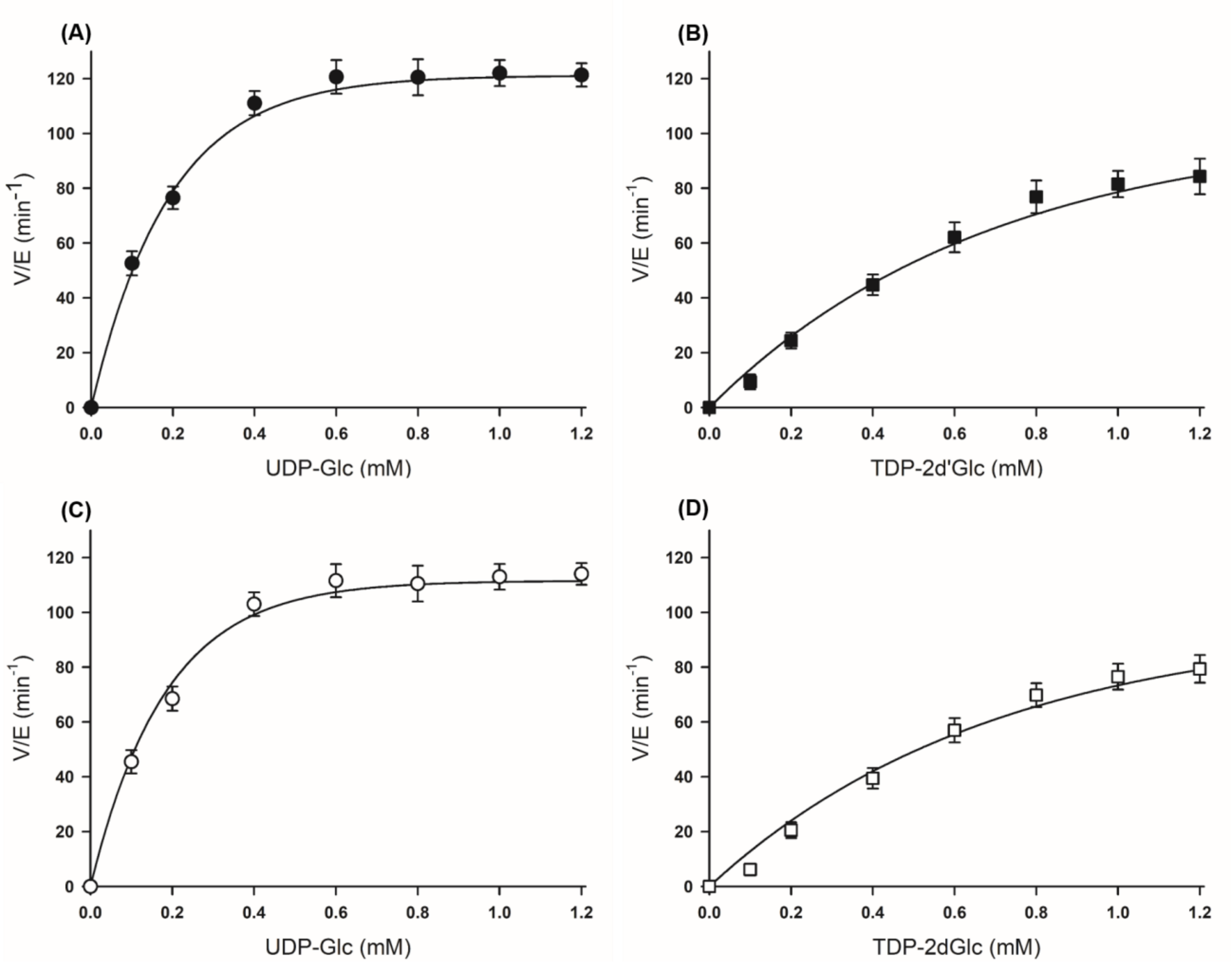

Supplementary Figure S1. Michaelis-Menten kinetics for recombinant StSPGT-catalyzed production of SP glycosides using the fixed glycosyl acceptor concentration with the varied glycosyl donor concentrations. (A) Reactions with hydroxyphenyl-2-propanol (HPP2) and uridine diphosphate-glucose (UDP-Glc). (B) Reactions with HPP2 and thymidine diphosphate-2-deoxy-glucose (TDP-2'dGlc). (C) Reactions with hydroxyphenyl-3-propanol (HPP3) and UDP-Glc. (D) Reactions with HPP3 and TDP-2'dGlc.

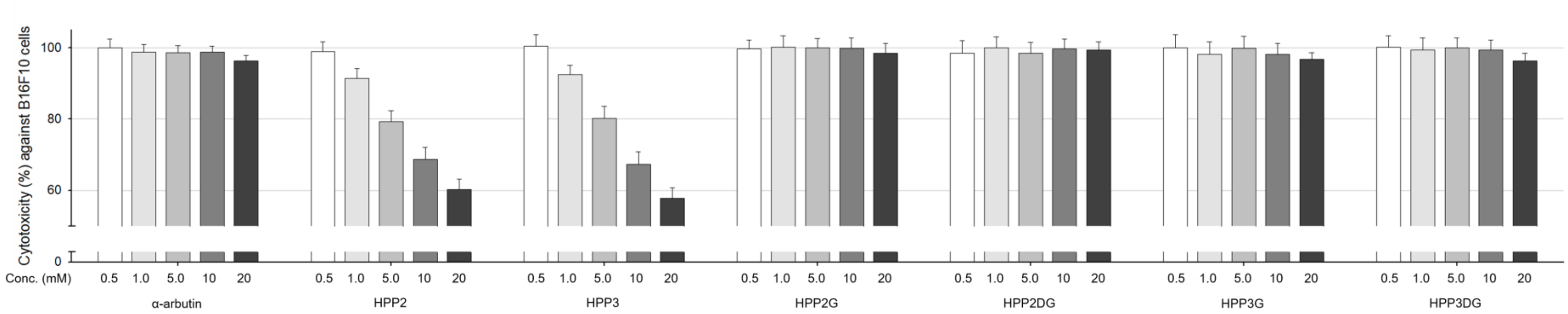

Supplementary Figure S2. Cytotoxicity of SP aglycones and their biosynthetic glycosides against B16F10 murine melanoma cells with varied concentrations (0.5, 1.0, 5.0, 10 and 20 mM). α-arbutin was utilized as a positive control. HPP2: hydroxyphenyl-2-propanol; HPP3: hydroxyphenyl-3-propanol; HPP2G: hydroxyphenyl-2-propanoyl-*O*-α-glucoside; HPP2DG: hydroxyphenyl-2-propanoyl-*O*-α-2''-deoxyglucoside; HPP3G: hydroxyphenyl-3-propanoyl-*O*-α-glucoside; HPP3DG: hydroxyphenyl-3-propanoyl-*O*-α-2''-deoxyglucoside.

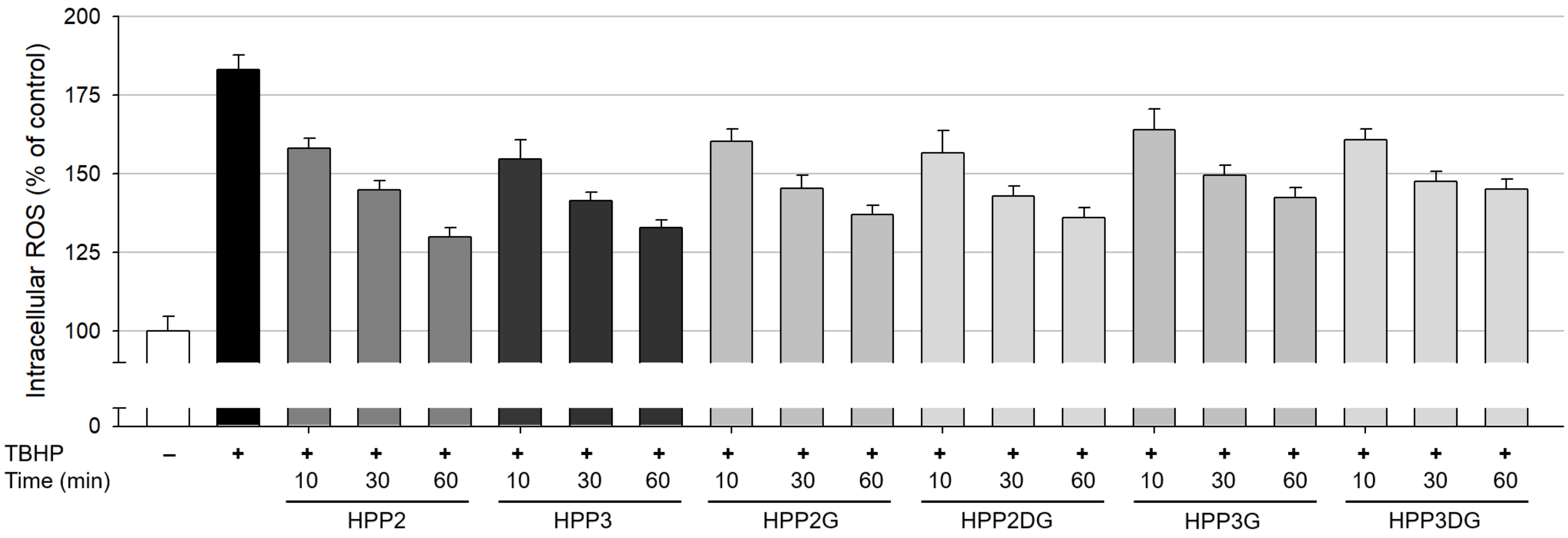

Supplementary Figure S3. The *in vivo* ROS scavenging antioxidant activity of SP aglycones and their biosynthetic glycosides against *tert*-butyl hydroperoxide (TBHP)-induced B16F10 cells at fixed 20 μM treatments with extended time intervals (10, 30 min and 1 h). HPP2: hydroxyphenyl-2-propanol; HPP3: hydroxyphenyl-3-propanol; HPP2G: hydroxyphenyl-2-propanoyl-*O*-α-glucoside; HPP2DG: hydroxyphenyl-2-propanoyl-*O*-α-2''-deoxyglucoside; HPP3G: hydroxyphenyl-3-propanoyl-*O*-α-glucoside; HPP3DG: hydroxyphenyl-3-propanoyl-*O*-α-2''-deoxyglucoside.

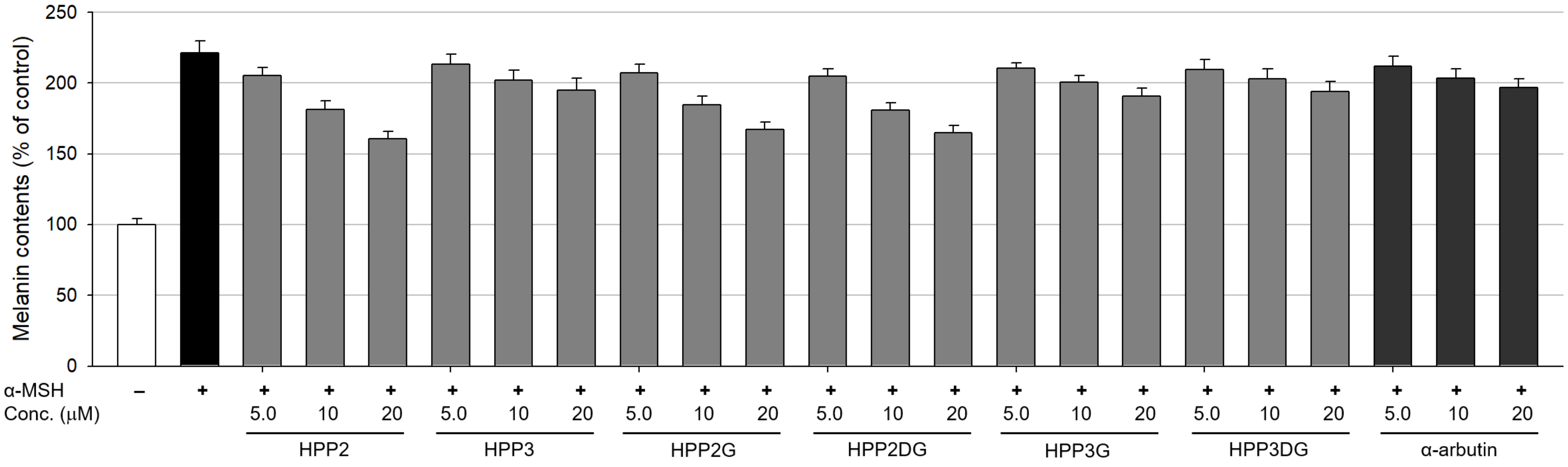

Supplementary Figure S4. The inhibitory activity of SP aglycones and their biosynthetic glycosides against the intracellular melanin biosynthesis in B16F10 cells with varied concentrations (5.0, 10 and 20 μM). α-arbutin was utilized as a positive control. HPP2: hydroxyphenyl-2-propanol; HPP3: hydroxyphenyl-3-propanol; HPP2G: hydroxyphenyl-2-propanoyl-*O*-α-glucoside; HPP2DG: hydroxyphenyl-2-propanoyl-*O*-α-2''-deoxyglucoside; HPP3G: hydroxyphenyl-3-propanoyl-*O*-α-glucoside; HPP3DG: hydroxyphenyl-3-propanoyl-*O*-α-2''-deoxyglucoside.
